# A retrospective evaluation of confirmed and suspected poisonings in 166 cats between 2016 and 2020

**DOI:** 10.14202/vetworld.2023.1940-1951

**Published:** 2023-09-23

**Authors:** Carina Markert, Romy Monika Heilmann, Dschaniena Kiwitz, René Dörfelt

**Affiliations:** 1 Department for Small Animals, Tierklinik Hofheim, 65719 Hofheim am Taunus, Germany; 2 Department for Small Animals, University of Leipzig, 04103 Leipzig, Germany; 3 Clinic of Small Animal Medicine, LMU Munich, 80539 Munich, Germany

**Keywords:** clinical signs, intoxication, outcome, seizures, toxicant

## Abstract

**Background and Aim:**

Poisonings commonly bring cats and dogs to veterinary emergency facilities. This retrospective study aimed to analyze clinical signs, confirmed or suspected toxicants, treatments, and outcomes of feline poisoning cases presented over 5 years to the emergency service of a small animal referral center.

**Materials and Methods:**

Medical records of 166 cats were evaluated for a history of confirmed or presumed poisoning, suspected toxicant, clinical signs, treatment, and outcome. Poisoning probability was determined using patient history, clinical findings, observation, toxicologic examination, and, in some cases, gastric contents.

**Results:**

Most cats were hospitalized (94.0%) due to poisoning with mostly unknown toxicants (48.2%), rodenticides (21.1%), and various toxic plants (12.0%), followed by antiparasitics (6.0%), chemicals (6.0%), drugs (4.2%), tetrahydrocannabinol (1.2%), or inhaled smoke (1.2%). Patients presented predominantly with neurologic deficits (68.7%), reduced general condition (60.2%), and hypothermia (43.4%). The survival rate was 88.6%. Most cats (93.2%) showed no apparent complications at the time of discharge from the hospital. Toxicant-related complications (48.2%) included thermodysregulation (22.9%), central nervous system signs (18.7%), respiratory issues (7.2%), nephrotoxicity (6.0%), gastrointestinal complications (4.8%), evidence of hepatic failure (4.8%), and hemorrhage (1.8%).

**Conclusion:**

In this study, the causative toxicant remained unidentified in many cases. Known poisonings were mostly caused by rodenticides. Neurological signs were the most common clinical presentation. Survival rates were high and comparable with those reported by others.

## Introduction

Poisonings commonly bring cats and dogs to veterinary emergency facilities [1]. Cats are conventionally considered less prone to poisoning than dogs; however, a rise in admissions of feline poisoning cases has been observed in recent years [2, 3]. Rodenticide ingestion has been the primary concern in cats and continues to be significant, with other toxicoses emerging in recent years [4–17].

Challenges in recognizing or confirming poisoning in cats are primarily the varying nonspecific clinical signs [13]. Therefore, diagnosing poisoning is often based on excluding other differentials. Neurological signs often dominate; however, the clinical sign severity in confirmed or suspected poisoning cases can vary from very mild and barely recognizable to overt presentations with fatal outcomes [18, 19]. The odds of survival primarily depend on the type and amount of toxicant and the timely initiation of treatment [19–21]. With few exceptions, any patient with suspected poisoning is to be treated as a medical emergency, requiring an initial clinical assessment followed by stabilization of the patient [21]. Supportive therapy, including early decontamination (e.g., bathing, induced emesis, gastric or rectal lavage, and activated charcoal) and elimination, is the cornerstone of treatment [6, 7, 21–29]. In particular, the rising incidence of accidental permethrin application warrants local decontamination measures [6]. Accidental drug toxicities are rising among indoor cats; therefore, intravenous (IV) lipid therapy has gained popularity for eliminating these substances [11, 30–33]. If the specific toxicant is known, specific antidote therapy should be used whenever possible and available [25, 34–36].

Given the frequency of cats being presented to veterinary emergency centers due to poisonings, providing an overview of the clinical presentation, treatments, and outcomes of confirmed or suspected poisonings in cats is warranted. Therefore, this study aimed to retrospectively evaluate a large cohort of cats with confirmed or suspected poisonings, including outcomes that were presented at the emergency service of a transregional veterinary referral center in Germany over 5 years.

## Materials and Methods

### Ethical approval

This study did not involve the use of animals; therefore, ethical approval was not specifically required for this study.

### Study period and location

The study was conducted from January 2016 to December 2020 at the Clinic for Small Animals “Tierklinik Hofheim”, Hofheim,Germany.

### Study design

Electronic medical records (MR) of the emergency service of a large veterinary referral center were searched for “poisoning/intoxication” and “cat” presented between 2016 and 2020. Out of 17,303 cats presented to the emergency service during the same period, 166 cases were identified and included in the retrospective evaluation.

Information retrieved from the MR were: (1) Signalment, (2) level of certainty of toxicant exposure, (3) clinical signs, (4) treatment by the owner or referring veterinarian before presentation, (5) clinical signs and physical examination findings at admission and during hospitalization, (6) the toxicant (confirmed or suspected) and duration from the time of exposure to appearance of clinical signs or admission to the clinic, (7) route of poisoning, and (8) clinicopathologic findings. Routine blood tests included a hematology (ProCyte Dx, Idexx, Kornwestheim, Germany), biochemistry profile (Integra 400 plus, Roche Cobas, Rotkreuz ZG, Switzerland), ionized calcium concentration (I-Stat, Scil, Viernheim, Germany), and clotting times (QuickVet, Scil, Viernheim, Germany). Furthermore, (9) treatment, including decontamination and elimination strategies, (10) outcome, and (11) disease course with any complications and duration of hospitalization were analyzed. For cats treated on an outpatient basis, the duration of treatment was inferred from the MR.

By integrating all available information from individual cats, the suspicion for poisoning was classified as either “certain,” “very likely,” or “probable.” The basis for assigning cats to this classification was, in descending order, “patient history,” “clinical signs,” “patient observation,” “toxicologic examination,” and “gastric contents,” yielding differentially weighed probabilities of poisoning for the cats included.

Poisonings were classified as “certain” if the ingestion of a specific toxicant was observed; the toxicant was detected on a toxicologic urine analysis (Forensic Toxicology Laboratory and Clinical Toxicology Laboratory, University of Göttingen, Germany) or, in select cases, if clinically and clinicopathologically evident. Cats were classified as “very likely” poisoned if showing typical clinical signs of the suspected poisoning and “probable” if the poisoning was possible and suspected by the attending veterinarian, given the clinical presentation of the cat; however, the patient history was not conclusive. Cats with primary neurological disorders were excluded from the study. In addition, cats were excluded if the owner solely presumed the poisoning without support from patient history, clinical signs, or clinicopathologic findings.

### Statistical analysis

Patient data were recorded using a commercial software (Excel, Microsoft Office 2016, Microsoft Corporation, Redmond, WA, USA) and were statistically analyzed (Prism 5, GraphPad, San Diego, CA, USA). Continuous data were evaluated for normal distribution using the D’Agostino and Pearson normality test, based on which summary statistics were reported as either mean ± standard deviation (parametric data) or median and range (nonparametric data). Possible associations between bradycardia and hypothermia at presentation with outcome (survival vs. non-survival) were assessed using Fisher’s exact test. Rectal body temperature and heart rates were compared between survivors and non-survivors using the Mann–Whitney U test. p < 0.05 was considered statistically significant.

## Results

### Signalment

Over the 5 years, 166 cats were presented with confirmed or suspected poisoning. Predominant breeds were Domestic Shorthair (124/166, 74.7%), British Shorthair (14/166, 8.4%), crossbreed (12/166, 7.2%), and Maine Coon (4/166, 2.4%). Over half of the cats with outdoor access showed clinical signs on returning from outside (46/83, 55.4%).

Male cats (69/166, 41.6% neutered and 14/166, 8.4% intact) were represented with similar frequency as female cats (63/166, 38.0% spayed and 19/166, 11.5% intact). The median age of all cats included in the study was 4.8 years (0.2–19.0 years), and the median weight was 4.1 kg (0.8–8.5 kg; Table-1).

**Table-1:** Signalment and time from confirmed or suspected toxicant ingestion or contact to toxicant to onset of clinical signs or hospital admission of 166 cats with signs of poisoning.

Parameter (unit)	Values	n
Age (years)	4.8 (0.2–19.0)	157
Weight (kg)	4.1 (0.8–8.5)	164
Time until clinical signs appeared (h)	3.0 (0.1–72.0)	64
Time until hospital admission (h)	6.0 (0.7–92.0)	62

n=number of cats (of all 166 cats in the study) from which the parameter was available in the medical records. Non-parametric data are presented as median (range)

### Patient history and clinical presentation

Clinical signs became apparent within a median of 3.0 h (0.1–72.0 h; n = 64) after suspected toxicant ingestion or contact, and presentation at the clinic was within a median of 6.0 h (0.7–92.0 h; n = 62). The most common clinical signs observed by the owners or the referring veterinarians were neurological signs (67.5%), followed by altered general condition (44.0%), and gastrointestinal signs (32.5%). Tachypnea (6.0%), hypothermia or hyperthermia (4.8%), hemorrhage (3.6%), cardiovascular compromise, and dehydration (1.8%) were less common (Tables-1 and 2).

**Table-2:** Clinical signs reported in the medical history and at the time of admission in 166 cats with confirmed or suspected poisoning.

Category	Clinical sign	Medical history (%)	Clinical examination (%)
Neurologic signs	Total*	112 (67.5)	114 (68.7)
	Hyperesthesia/tremor	51 (30.7)	56 (33.7)
	Focal seizures	16 (9.6)	29 (17.5)
	Seizures	13 (7.8)	18 (10.8)
	Status epilepticus	3 (1.8)	7 (4.2)
	Ataxia	50 (30.1)	29 (17.5)
	Behavioral changes	27 (16.3)	27 (16.3)
	Ocular changes	7 (4.2)	30 (18.1)
	Miosis	3 (1.8)	15 (9.0)
	Mydriasis	5 (3.0)	23 (13.9)
	Peripheral nerve signs	4 (2.4)	4 (2.4)
Altered general condition	Total*	73 (44.0)	100 (60.2)
	Mildly reduced condition	29 (17.5)	25 (15.1)
	Moderately reduced condition	26 (15.7)	30 (18.1)
	Markedly reduced condition	18 (10.8)	33 (19.9)
	Excitement	3 (1.8)	11 (6.6)
	Pain	3 (1.8)	8 (4.8)
Gastrointestinal signs	Total*	54 (32.5)	17 (10.2)
	Vomiting/nausea	35 (21.1)	2 (1.2)
	Salivation	17 (10.2)	13 (7.8)
	Diarrhea	12 (7.2)	3 (1.8)
	Anorexia	16 (9.6)	-
	Polyphagia	1 (0.6)	-
	Oral mucosa lesions	-	5 (3.0)
Cardiovascular/hydration changes	Total*	3 (1.8)	68 (41.0)
	Bradycardia (<160/min)	1 (0.6)	37 (22.3)
	Tachycardia (>200/min)	-	12 (7.2)
	Arrhythmia	1 (0.6)	1 (0.6)
	Pale mucous membranes	-	4 (2.4)
	Hyperemic mucous membranes	-	2 (1.2)
	Prolonged CRT	-	1 (0.6)
	Pulse deficit	-	1 (0.6)
	Hypotension	1 (0.6)	-
	Dehydration	1 (0.6)	27 (16.3)
Altered thermoregulation	Total*	8 (4.8)	84 (50.6)
	Hypothermia (<38.0°C; <100.4°F)	6 (3.6)	72 (43.4)
	Hyperthermia (>39.0°C; >102.2°F)	2 (1.2)	12 (7.2)
Respiratory signs	Total*	10 (6.0)	22 (13.3)
	Increased RR (>30/min)	7 (4.2)	16 (9.6)
	Dyspnea	3 (1.8)	6 (3.6)
Hemorrhage	Total*	6 (3.6)	8 (4.8)
	Epistaxis	1 (0.6)	-
	Hematuria	1 (0.6)	1 (0.6)
	Oral hemorrhage	1 (0.6)	1 (0.6)
	Hematemesis	1 (0.6)	-
	Hematoma	2 (1.2)	3 (1.8)
	Hemothorax	1 (0.6)	1 (0.6)
	Anemic mucous membranes	1 (0.6)	5 (3.0)
	Mucosal lesions	-	1 (0.6)
	Hemo-abdomen	-	1 (0.6)

* Refers to the number of cats to which at least one of the clinical signs within that category was seen. Multiple entries for individual cats within a category of clinical signs are possible. CRT=capillary refill time, RR=Respiratory rate

Clinical signs at the time of hospital admission were predominantly neurological signs (68.7%), altered general condition (60.2%), altered thermoregulation (50.6%), cardiovascular signs or altered hydration status (41.0%), tachypnea (13.3%), gastrointestinal signs (10.2%), or hemorrhage (4.8%). Before admission, one cat (0.6%) showed no clinical signs of poisoning, according to the owner, and the physical examination at admission was unremarkable in nine (5.4%) cats (Table-2).

### Physical examination findings

Initial heart rates were 172 ± 34/min (n = 145). Bradycardia (n = 37; 25.5%) and tachycardia (n = 12; 8.3%) were infrequently observed. Respiratory rates were 57 ± 40/min (n = 20; 12%). Tachypnea (n = 16; 80.0%); however, not bradypnea, was reported. The median rectal temperature was 38.1°C [100.6°F] (n = 161). Hypothermia was reported more frequently (n = 72; 44.7%) than hyperthermia (n = 12; 7.5%; Table-3).

**Table-3:** Physical examination parameters of 166 cats with confirmed or suspected poisoning at presentation.

Parameter (unit)	Reference interval	Values	n
Heart rate (beats/min)	160–200	172±34	145
Respiratory rate (breaths/min)	<30	57±40	20
Rectal temperature			
(°C)	38.0–39.0	38.1 (32.3–41.8)	161
(°F)	100.4–102.2	100.6 (90.1–107.2)	

n=Number of cats (of all 166 cats in the study) from which the parameter was available in the medical records. Normally distributed data are presented as mean±standard deviation; nonparametric data are presented as median (range)

### Clinicopathologic findings

Anemia was detected in 17.4% (n = 23) of the cats, increased serum creatinine concentration in 11.0% (n = 14), and increased urea in 16.0% (n = 20). More cats were hyperglycemic (n = 48; 37.2%) than hypoglycemic (n = 10; 7.8%). Hypokalemia (n = 21; 16.9%) and hyperkalemia (n = 6; 4.8%) were observed, as were hyponatremia (n = 16; 12.7%) and hypernatremia (n = 3; 2.4%). Coagulation times were prolonged in 12/13 cats (92.3%). Clinically relevant increases (>20% above reference interval) in the activated partial thromboplastin time (aPTT) were observed in 69.2% of these cats (n = 9) with values beyond the analytical range of the instrument in 53.8% (n = 7). Prothrombin time (PT) was also clinically relevantly increased above the upper reference limit in seven cats (n = 7; 53.9%; Table-4).

**Table-4:** Laboratory findings (hematology, clinical chemistry, and clotting times) in 166 cats with confirmed or suspected poisoning at presentation.

Parameter (unit)	Reference interval	n (%)	Mean (range)	Decreased (%)	Increased (%)
HCT (%)	30.3–52.3	132 (79.5)	36.9 (8.2–55.1)	23/132 (17.4)	2/132 (1.5)
WBC (×10^9^/L)	2.87–17.02	131 (78.9)	8.9 (0.7–22.3)	5/131 (3.8)	7/131 (5.3)
Plt (×10^9^/L)	152–600	131 (78.9)	268 (32–701)	18/131 (13.7)	3/131 (2.3)
TP (g/L)	57–90	136 (81.9)	72.0 (47.0–101.0)	5/136 (3.7)	2/136 (1.5)
Alb (g/L)	23–45	136 (81.9)	35.0 (21.0–47.8)	1/136 (0.7)	2/136 (1.5)
Crea (μmol/L)	0–167	128 (77.1)	103 (44–2420)	-	14/128 (10.9)
Urea (mmol/L)	5.2–12.5	125 (75.3)	9.3 (4.3–80.1)	3/125 (2.4)	20/125 (16.0)
Gluc (mmol/L)	5–7.4	129 (77.7)	6.5 (4.3–21.5)	10/129 (7.8)	48/129 (37.2)
AST (nkat/L)	0–90	43 (25.9)	42 (16–2093)	-	8/43 (18.6)
ALT (nkat/L)	0–1,733	87 (52.4)	59 (17–311)	-	9/87 (10.3)
AP (nkat/L)	0–950	37 (22.3)	37 (16–105)	-	7/37 (18.9)
Bili (μmol/L)	0–7	78 (47.0)	0.7 (0.0–19.0)	-	2/78 (2.6)
Na (mmol/L)	147–160	126 (75.9)	152 (129–161)	16/126 (12.7)	3/126 (2.4)
K (mmol/L)	3.4–5.4	124 (74.7)	3.7 (2.4–9.5)	21/124 (16.9)	6/124 (4.8)
Cl (mmol/L)	104–120	126 (75.9)	116 (78–228)	4/126 (3.2)	21/126 (16.7)
Ca (mmol/L)	2.2–2.98	98 (59.0)	2.7 (2.0–3.3)	3/98 (3.1)	6/98 (6.1)
Ca^++^ (mmol/l)	1.12–1.4	3 (1.8)	1.37 (1.33–1.54)	-	1/3 (33.3)
*aPTT (s)	≤105	13 (7.8)	>200 (102–>200)	-	12/13 (92.3)
**PT (s)	≤19	13 (7.8)	>35 (16.8–>35)	-	7/13 (53.8)

* Measuring range up to 200 s, with values above that range reported as “>200 s.”

** Measuring range up to 35 s, with values above that range reported as “>35 s.” n=Number of cats (of all 166 cats in the study) in which the respective parameter was recorded. HCT=Hematocrit, WBC=Leukocyte (white blood cell) count, Plt=Platelet count, TP=Total protein concentration, Alb=Albumin concentration, Crea=Creatinine concentration, Urea=Urea concentration, Gluc=Glucose concentration, AST=Aspartate aminotransferase activity, ALT=Alanine aminotransferase activity, AP=Alkaline phosphatase activity, Bili=Bilirubin concentration, Na=Sodium concentration, K=Potassium concentration, Cl=Chloride concentration, Ca=Calcium concentration, Ca^++^=Ionized calcium concentration, aPTT=Activated partial thromboplastin time, PT=Prothrombin time

### Treatment

Treatment before hospital admission by the owner or referring veterinarian was reported in 37/166 cats (22.3%). Decontamination (3.6%), including bathing by the owner (n = 5; 3.0%), administration of charcoal (n = 1; 0.6%), or toxicant elimination (1.2%) by the referring veterinarian, was rarely attempted. Pretreatment performed by the referring veterinarian (with one exception) consisted of anticonvulsants (6.6%), corticosteroids (6.0%), antimicrobials (5.4%), analgesics (4.8%), including non-steroidal anti-inflammatory drugs (NSAIDs; 4.2%), fluid therapy (4.2%), antiemetics (3.0%), B vitamins (2.4%), sedatives (1.2%), atropine (1.2%), supplemental glucose (1.2%; oral glucose given by the owner to one cat), and anesthetics and other medication (each 0.6%; Tables-5 and 6).

**Table-5:** Decontamination and elimination measures before and after hospital admission of 166 cats with confirmed or suspected poisoning.

Decontamination	Measure	Before admission (%)	After admission (%)
	Total*	6 (3.6)	58 (34.9)
	Emesis (dexmedetomidine)	-	4 (2.4)
	Emesis (xylazine)	-	3 (1.8)
	Bathing	5 (3.0)	9 (5.4)
	Gastric lavage	-	6 (3.6)
	Rectal lavage	-	3 (1.8)
	Oral lavage	-	6 (3.6)
	Laxative (lactulose)	-	6 (3.6)
	Adsorbent (charcoal)	1 (0.6)	34 (20.5)

**Elimination**	**Measure**	**Before admission**	**After admission**

	Total*	2 (1.2)	103 (62.0)
	Enhanced diuresis (furosemide)	2 (1.2)	5 (3.0)
	Enhanced diuresis (mannitol)	-	3 (1.8)
	Intravenous lipid infusion	-	100 (60.2)

* Refers to the number of cats to which at least one of the decontamination or elimination methods was undertaken. Multiple entries for individual cats are possible.

**Table-6:** Treatment before and after hospital admission of 166 cats with confirmed or suspected poisoning.

Drug group	Active drug	Before admission (%)	After admission (%)
Isotonic intravenous fluids	Total*	7 (4.2)	156 (94.0)
Anticonvulsants	Total*	11 (6.6)	54 (32.5)
	Diazepam	11 (6.6)	52 (31.3)
	Phenobarbital	-	32 (19.3)
	Levetiracetam	-	2 (1.2)
Antiemetics	Total*	5 (3.0)	40 (24.1)
	Maropitant	4 (2.4)	36 (21.7)
	Metoclopramide	1 (0.6)	6 (3.6)
	Dimenhydrinate	-	1 (0.6)
Gastric protectants	Total*	-	23 (13.9)
	Pantoprazole	-	22 (13.3)
	Sucralfate	-	10 (6.0)
Sedatives	Total*	2 (1.2)	18 (10.8)
	Dexmedetomidine	-	10 (6.0)
	Medetomidine	1 (0.6)	-
	Butorphanol	-	7 (4.2)
	Ketamine	-	1 (0.6)
	Unknown	1 (0.6)	-
Analgesics	Total*	8 (4.8)	14 (8.4)
NSAIDs	Meloxicam	4 (2.4)	5 (3.0)
	Tolfenamic acid	2 (1.2)	4 (2.4)
	Unknown	1 (0.6)	-
Opioids	Buprenorphine	1 (0.6)	4 (2.4)
	Fentanyl	-	3 (1.8)
Corticosteroids	Total*	10 (6.0)	-
Anesthetics	Total*	1 (0.6)	10 (6.0)
	Propofol	1 (0.6)	10 (6.0)
	Pentobarbital	1 (0.6)	4 (2.4)
Potassium substitution	Potassium chloride	-	10 (6.0)
Antidots (coumarin derivatives)	Vitamin K_1_	-	8 (4.8)
Antimicrobials	Total*	9 (5.4)	6 (3.6)
	Amoxicillin+clavulanic acid	3 (1.8)	5 (3.0)
	Cefovecin	1 (0.6)	-
	Marbofloxacin	1 (0.6)	1 (0.6)
	Enrofloxacin	1 (0.6)	-
	Unknown	3 (1.8)	-
Supplemental oxygen	Total*	-	6 (3.6)
Glucose	Total*	2 (1.2)	2 (1.2)
CPR	Total*	-	2 (1.2)
Atropine (due to bradycardia)	Total*	2 (1.2)	2 (1.2)
Saline (0.9%) inhalation	Total	-	2 (1.2)
Others	Total*	8 (4.8)	21 (12.7)
	Atipamezole	-	5 (3.0)
	Mirtazapine	-	3 (1.8)
	Probiotics	-	3 (1.8)
	Moisturizing eye drops	-	2 (1.2)
	Blood transfusion	-	2 (1.2)
	B vitamins	4 (2.4)	2 (1.2)
	Barium sulfate	-	2 (1.2)
	Sotalol	-	1 (0.6)
	Propentophylline	-	1 (0.6)
	Deworming	1 (0.6)	-
	Amlodipine	1 (0.6)	-
	Terbutaline	1 (0.6)	-
	Metamizole	1 (0.6)	-
	Butylscopolamine + metamizole	1 (0.6)	-

* Refers to the number of cats to which at least one of the drugs within that drug group was administered. Multiple entries for individual cats within a drug group are possible. NSAIDs=Nonsteroidal anti-inflammatory drugs, CPR=Cardiopulmonary resuscitation

Fifty-eight cats underwent decontamination at the time of admission (34.9%), including charcoal administration (20.5%; as monotherapy in 28 cats, 16.9%), bathing (5.4%), induced emesis (4.2%) and gastric (3.6%), oral (3.6%), or rectal lavage (1.8%). Emesis induction with dexmedetomidine (4/7, 57.1%) and/or xylazine (3/7, 42.9%) was successful in five cats (71.4%). Toxicant elimination by forced diuresis (4.2%) with furosemide (3.0%) or mannitol (1.8%) and IV lipid infusion (60.2%) was attempted in 103 cats (62%). Cats treated with IV lipid therapy had encountered poisoning with alpha-chloralose (α-chloralose) (n = 16), didecyldimethylammonium chloride (n = 5), other rodenticides and permethrin (n = 4), robenacoxib and tetrahydrocannabinol (THC) (n = 2) or not exactly known toxicants (n = 58), among other toxicants (n = 1).

Most cats received IV fluid therapy (94.0%). Anticonvulsants were administered to every third cat (32.5%) and antiemetics to every fourth cat (24.1%). Gastroprotective agents (13.9%), sedatives (10.8%), and analgesics (8.4%) were also administered. Few cats were anesthetized (6.0%) or received supplemental potassium (6.0%) or Vitamin K_1_ as an antidote to coumarin derivatives (4.8%). Six cats (3.6%) received antimicrobials or other treatment, including oxygen therapy (3.6%), the α_2_-antagonist atipamezole (3.0%) to reverse sedation, glucose substitution (1.2%), saline inhalation (1.2%), or atropine to address bradycardia (1.2%). Cardiopulmonary resuscitation was performed in two cats (1.2%); both were unsuccessful. Treatment was rejected by the owners in two cases (1.2%; Tables-5 and 6).

### Toxicants and route of poisoning

Causative toxicants assumed based on patient history (44.6%), clinical signs (40.4%), observation of toxicant ingestion (11.4%), results of toxicological analyses (2.4%), and/or toxicant detection in gastric contents (1.2%) led to the classification as “certain poisoning” (22.9%), “very likely poisoning” (42.8%), or “probable poisoning” (34.3%). Toxicologic analysis confirmed α-chloralose poisoning in four cats (4/5, 80%; Table-7).

**Table-7:** Classification of the 166 cats presented due to confirmed or suspected poisonings, based on the level of certainty to classify cases as certain, very likely, or probable poisoning.

Basis of classification	Level of certainty for an intoxication			

	All (%)	Certain (%)	Very likely (%)	Probable (%)
All		38/166 (22.9)	71/166 (42.8)	57/166 (34.3)
Patient history	74/166 (44.6)	7/38 (18.4)	44/71 (62.0)	23/57 (40.4)
Clinical signs	67/166 (40.4)	7/38 (18.4)	26/71 (36.6)	34/57 (59.6)
Observation	19/166 (11.4)	19/38 (50.0)	-	-
Toxicological examination	4/166 (2.4)	4/38 (10.5)	-	-
Gastric content	2/166 (1.2)	1/38 (2.6)	1/71 (1.4)	-

Toxicant exposure was mostly through ingestion (132/166, 79.5%), followed by transdermal (9/166, 5.4%), transdermal and oral (2/166, 1.2%), or inhalation (2/166, 1.2%). The route of exposure was not specified in 21 cats (12.7%).

A total of 40 different toxicants were recorded; however, the toxicant remained unknown in 80 cats (48.2%). Rodenticides were most commonly reported (21.1%), including α-chloralose (13.3%) and coumarin derivatives (5.4%). Ingested toxic plants (12.0%) included *Lilium* species (3.6%) and other saponin-containing plants (2.4%). Antiparasitics caused poisoning in ten cats (6.0%), with permethrin (3.6%) being the most common agent. Chemical toxicants were reported in ten cats (6.0%) and medication-associated poisonings in seven cats (4.2%), including NSAIDs (4/166, 2.4%). Tetrahydrocannabinols (2/166, 1.2%) and smoke inhalation affected two cats (1.2%; Table-8).

**Table-8:** Suspected or confirmed toxicants in 166 cats presented with clinical signs or a history of poisoning and the corresponding outcomes.

Toxicant group	Toxicant	Cats (%)	Outcomes		

			Survival (%)	Euthanasia (%)	Death (%)
All		166	147 (88.6)	16 (9.6)	3 (1.8)
Unknown	Total	80 (48.2)	72/80 (90.0)	6/80 (7.5)	2/80 (2.5)
Rodenticides	Total	35 (21.1)	31/35 (88.6)	4/35 (11.4)	-
	Alpha(α-) chloralose	22 (13.3)	20	2	-
	Coumarin derivatives	9 (5.4)	8	1	-
	Others	4 (2.4)	3	1	-
Plants and plant extracts	Total	20 (12.0)	15/20 (75.0)	5/20 (25.0)	-
	Lilies (mostly *Lilium* species or unknown)	6 (3.6)	3	3	-
	Saponin-containing plants (e.g., *Dracaena draco, Cyclamen persicum*)	4 (2.4)	4	-	-
	*Monstera deliciosa*	2 (1.2)	2	-	-
	Mushroom (*Agaricus campestris*)	1 (0.6)	1	-	-
	*Azadirachta indica/Eucalyptus* species	1 (0.6)	1	-	-
	*Hedera helix*	1 (0.6)	-	1	-
	*Physalis*	1 (0.6)	1	-	-
	*Spathiphyllum*	1 (0.6)	1	-	-
	*Melaleuca alternifolia* (oil)	1 (0.6)	1	-	-
	*Geranium* species	1 (0.6)	1	-	-
	*Allium cepa*	1 (0.6)	-	1	-
Antiparasitics	Total	10 (6.0)	8/10 (80.0)	1/10 (10.0)	1/10 (10.0)
	Permethrin (single active ingredient)	4 (2.4)	2	1	1
	Pyrethrin	2 (1.2)	2	-	-
	Milbemycin	1 (0.6)	1	-	-
	Permethrin + imidacloprid	1 (0.6)	1	-	-
	Permethrin + pyriproxyfen+dinotefuran	1 (0.6)	1	-	-
	Moxidectin + imidacloprid	1 (0.6)	1	-	-
Chemicals	Total	10 (6.0)	10/10 (100)	-	-
	Didecyldimethylammonium chloride	5 (3.0)	5	-	-
	Fertilizer	2 (1.2)	2	-	-
	Cleaning solution	1 (0.6)	1	-	-
	Paint	1 (0.6)	1	-	-
	Iron (heat pad)	1 (0.6)	1	-	-
Medication	Total	7 (4.2)	7/7 (100)	-	-
NSAID	Total	4 (2.4)	4	-	-
	Ibuprofen	2 (1.2)	2	-	-
	Robenacoxib	2 (1.2)	2	-	-
Other	Lisdexamfetamine	1 (0.6)	1	-	-
	DL-methionine	1 (0.6)	1	-	-
	Terbutaline	1 (0.6)	1	-	-
Illegal drugs	Tetrahydrocannabinol	2 (1.2)	2/2 (100)	-	-
Smoke inhalation	Smoke	2 (1.2)	2/2 (100)	-	-

NSAID=Nonsteroidal anti-inflammatory drug

### Outcome

Ten cats (6.0%) were treated on an outpatient basis, whereas most cats (94.0%) were hospitalized (median: 2.0 days, range: 0.1–5.0 days). Approximately 89.0% (147/166) of the cats survived to discharge from the hospital, and 16 cats (9.6%) were euthanized. Toxicants in these cats included an unknown toxicant (37.5%), *Lilium* species (18.8%), α-chloralose (12.5%), permethrin, *Allium cepa*, coumarin derivatives, other rodenticides, or poisoning with *Hedera helix* (each 6.3%). Three cats (1.8%) died (unknown toxicant n = 2; permethrin n = 1; Table-8).

Most survivors (137/147, 93.2%) had no apparent complications, and more than half of these cats (85/147, 57.8%) were fully recovered at hospital discharge. Marked clinical signs developed or significant improvement failed in ten cats (6.8%), which were discharged early at the owners’ request and showed postictal changes (staggering, central blindness or impaired vision, and inability to walk), anorexia, or persistent marked azotemia.

Half of the cats with *Lilium* species poisoning (n = 3) developed acute kidney injury (AKI). In one of these cats, azotemia was progressive (serum creatinine increased from 1400 to 2365 μmol/L), whereas in the other cats, it was slightly regressive (2095–1833 μmol/L), or only one creatinine measurement was performed (2400 μmol/L). All three cats died, whereas the cats with low-grade azotemia (serum creatinine up to 207 μmol/L) survived.

Two-thirds of cats with permethrin poisoning (n = 4) survived. One cat experienced recurrent seizures after weaning from sedation and died. The other cat was euthanized after 34 h of sedation, anesthesia, and ventilation.

Bradycardia on presentation was not significantly more common in non-survivors (8/18; 44.4%) than survivors (29/127; 22.8%; p = 0.079), and heart rate did not differ significantly between non-survivors (median, 160 beats/min; range, 88–260/min) and survivors (median, 180 breaths/min; range, 92–300/min; p = 0.062). Rectal body temperature was significantly lower in non-survivors (median, 37.0°C/98.6°F; range, 32.3–40.6/90.2–105.1) compared to survivors (median, 38.1°C/100.6°F; range, 35.4–41.8/95.7–107.2; p = 0.002); however, hypothermia was not significantly more common in non-survivors (11/20; 55.0%) than survivors (61/142; 43.0%; p = 0.231).

Respiratory rates could not be compared between these groups due to the small number of cats, for which the respiratory rate at presentation was documented (Table-9).

**Table-9:** Outcome related to physical examination findings at presentation in 166 cats with confirmed or suspected poisoning.

Parameter (unit)	Reference interval	All	Survivors		Non-survivors		p-value
	
			Values	n	Values	n	
Heart rate (/min)	160–200	180 (88–300)	180 (92–300)	128	160 (88–260)	17	0.062
Respiratory rate (/min)	16–30	38 (16–120)	38 (16–120)	19	120	1	x
T (°C)	38.0–39.0	38.1 (32.3–41.8)	38.1 (35.4–41.8)	142	37.0 (32.3–40.6)	19	0.002

Non-parametric data are presented as median (range). x=No p*-*value reported due to the limited number of patients, T=Temperature, p*=*Significance level; statistical significance was set at p *<* 0.05.

### Complications

Toxicant-related complications occurred in almost half of the hospitalized cats (n = 80; 48.2%) and included mostly temperature (22.9%), central nervous system (18.7%), respiratory (7.2%), renal (6.0%), gastrointestinal and hepatic complications (4.8% each), or hemorrhage (1.8%; Table-10).

**Table-10:** Toxicant-associated complications in 80 of 166 cats (48.2%) with confirmed or suspected poisoning.

Organ system n (cats) (%) Toxicant	Temperature	CNS	Respiratory	Renal	GIT	Hepatic	Hemorrhage
						
	38 (22.9%)	31 (18.7%)	12 (7.2%)	10 (6.0%)	8 (4.8%)	8 (4.8%)	3 (1.8%)
Unknown (30)	*Hypothermia (15)	Persisting seizures (18) and Postictal coma (1)	Hypoxemia (4) Apnea (2) NPE (1) Bradypnea (1) Dyspnea (1)	AKI (2)	Vomiting (1)	Liver failure (1)	
α-chloralose (22)	Hypothermia (9)	Persisting seizures (11) and postictal coma (1)		AKI (1)	Vomiting (1)		
Coumarin derivative (6)	*Hypothermia (5)		Dyspnea (2)	AKI (1)		Liver failure (1)	Hemothorax (1) Hemo-abdomen (1)
Didecyldimethylammonium chloride (5)	*Hyperthermia (5)				Tongue erosions (5)		
*Lilium* species (4)				*AKI (3)		Liver failure (4)	
Permethrin (3)		Persisting seizures and postictal coma (1)	Apnea (1)			Liver failure (1)	
Other rodenticides (2)		Persisting seizures and postictal coma (1)			Vomiting (1)		
Ibuprofen (2)				AKI (1)			GIT hemorrhage (1)
Saponin-containing plants (1)	Hypothermia (1)					*Liver failure (1)	
*Monstera deliciosa* (1)				AKI (1)			
*Hedera helix* (1)	Hypothermia (1)						
*Spathiphyllum* (1)	Hyperthermia (1)						
*Allium cepa* (1)				AKI (1)			
Fertilizer (1)	Hypothermia (1)						

* Multiple mentions of a complication in the same patient belonging to the respective toxic substance. AKI=Acute kidney injury, α=Alpha, CNS=Central nervous system, GIT=Gastrointestinal, n=Number of cats, NPE=Neurogenic pulmonary edema

Ingestion of toxicants led to hypothermia (n = 32; 19.3%; unknown toxicant: n = 15, α-chloralose: n = 9, coumarin derivatives: n = 5) or hyperthermia (n = 6; 3.6%; predominantly didecyldimethylammonium chloride: n = 5). Two of five cats necessitating general anesthesia to control seizures had to be ventilated (n = 1 each for permethrin and unknown toxicant), and another cat died due to apnea (n = 1 unknown toxicant). Postictal comatose state without sedatives was observed in four cats (2.0%; n = 1 each for α-chloralose, permethrin, other rodenticide, and unknown toxicant), and seizures persisted in 31 cats (18.7%; unknown toxicant: n = 18, α-chloralose: n = 11, other rodenticide or permethrin: n = 1 each). Progressive kidney injury was detected by increasing serum creatinine concentration within the high-normal reference interval in five cats (5/24, 21.0%; n = 1 each for ibuprofen, a coumarin derivative, α-chloralose, *Monstera deliciosa*, and an unknown toxicant) and above the reference interval in two cats (8.0%; n = 1 each for *Lilium* species and unknown toxicant). Oliguric or anuric AKI was seen in three cats (n = 2 for *Lilium* species, n = 1 for *Allium cepa*).

Treatment-associated complications after IV lipid administration occurred in 5/166 (3.0%) cats. These complications included phlebitis after paravenous leakage of IV lipid infusion (n = 2), resulting in transient hyperthermia (n = 1), neurologic deficits with a semicomatose state (n = 1), and somnolence linked to bradycardia (n =1), as well as marked bradycardia (n = 1) or tachypnea (n = 1).

## Discussion

This study included 166 cats with confirmed or presumed poisoning that was presented over 5 years. To the best of our knowledge, this reports the largest cohort of feline poisoning cases within a short period [35].

Reasons for hospital admission based on patient history and clinical presentations at hospital admission were largely consistent and comprised mostly neurologic signs or an altered general condition. Neurologic signs are common with poisonings [5, 7, 12, 36]. Moreover, witnessing seizures alarms many owners to seek immediate veterinary care. Exhibiting reduced general condition is less specific and can occur with any disease. Cats were also often presented for gastrointestinal signs; however, evidence of altered thermoregulation, cardiovascular, and hydration status predominated on physical examination. A previous review of 138 feline poisoning cases reported similar presentations of neurologic, gastrointestinal and respiratory clinical signs, reduced general condition, and bleeding; however, clinical signs based on medical history were not differentiated from those at admission [35]. A possible explanation for more cats showing neurologic clinical signs in this study could be a higher rate of neurotoxic substance intake. However, interpreting clinical signs by medical laypersons versus the veterinary care team and collecting short patient histories during emergency consultations could introduce bias. Furthermore, temporal aspects must be considered as clinical signs may worsen or improve from their onset to hospital admission.

Approximately one-quarter of the cats showed bradycardia or tachycardia, likely due to increased vagal or sympathetic tone associated with the toxicant or specific drug [11, 13, 35]. Tachypnea can also be stress-related or linked to dyspnea. Many hypothermic patients presented intra- or postictally (57.0%) or in advanced shock (28%), and mild hypoglycemia (4.3–4.9 mmol/L) were observed, particularly with rodenticide poisoning (2%). Hypothermia was also detected in cats with comatose states (13%). Only one-third of the hyperthermic cats experienced seizures. More than half of the euthanized cats (56%) and two-thirds of the cats that died were hypothermic. Only one deceased patient was hypothermic. Finally, hypothermia rates were not different between survivors and non-survivors, contrasting with the results of a study that examined one specific toxicant group (organophosphates) [36]. Similar to that study, rectal body temperature was lower in non-survivors than in survivors in this study.

Our data indicate that poisonings primarily impact juvenile and young adult cats, possibly attributed to heightened curiosity behavior and associated elevated risk of ingesting foreign substances. However, the causative toxicant could not be determined in almost half of the patients, possibly due to a lower likelihood of witnessing toxicant ingestion in outdoor cats (with a prevalence of 50% in this study) and limited toxicological screenings. Another investigation reported the detection of the toxicant in a larger proportion of patients (80.4%) that comprised only one-third of outdoor cats, and poisonings were significantly more often classified as “certain” than the more conservative classification in our study (72% vs. 23%) [35]. Moreover, other studies included cats witnessed to have ingested the toxicant; however, without specifying the level of certainty or subcategories [13, 37]. Poisonings with ectoparasite prophylactics have been shown to cluster in cats [6, 7, 18, 32, 33, 38–40]. This involves primarily the accidental administration of spot-on formulations that are labeled only for dogs and are highly toxic to cats, where accidental oral administration by the owner or ingestion by the cat during grooming can cause neurotoxicity [38]. Two-thirds of the permethrin-poisoned cats in the present study survived (4/6; 67%), and survival rates for other toxicants ranged from 63% to 98% [7, 39, 40].

Almost a third of all anemic cats (hematocrit [HCT] <30.3%; median age 5.1 years) in our study were affected by poisoning with coumarin derivates, suggesting (mostly occult) bleeding. Furthermore, chronic kidney disease (CKD, n = 2) was considered to be an important contributor to anemia in these cats, and a small deviation (<1%) from the lower reference limit for HCT was interpreted as an age-related physiological finding in juvenile cats (n = 3). Increased serum creatinine concentrations (>167 μmol/L) in about 10% of the cats suggested direct kidney injury (two-thirds of the cats with *Lilium* species poisoning were azotemic), shock after dehydration, or CKD. Serial creatinine measurements performed in some cats revealed progressive azotemia (8%) or relevant increases in serum creatinine levels within the high-normal range (21%). Survival of *Lilium* species-poisoned cats with AKI was 50%. The previous study has shown a remarkably good prognosis with up to 100% survival following decontamination and IV infusion within 48 h without AKI development [37]. As seen in many cats, hyperglycemia can be explained by the physiological stress response. Hypoglycemia suggests severe cardiovascular compromise (e.g., sepsis) and/or consumption of glycogen stores (e.g., post-seizures); however, preanalytic effects cannot be definitively excluded. Prolongation of PT and aPTT was exclusively detected in coumarin derivative poisoning, whereas an isolated aPTT prolongation was also observed. Possible explanations for the increased aPTT are hepatopathy, inflammatory reaction, or disseminated intravascular coagulation. Significant alterations in laboratory parameters are rarely associated with poisoning.

Decontamination measures were performed less often (35%) than in a recent study [37]. As the initial emergency action, decontamination must be carefully balanced against potential risks and contraindications for each patient [21, 27, 41]. Decontamination is unhelpful or contraindicated for remote toxicant ingestion, absence of toxic effects, and in patients with impaired consciousness, seizures, compromised swallowing, or other aspiration pneumonia risk factors. Unlike non-volatile oily substances, emesis should not be induced for foaming, corrosive, sharp objects, or volatile hydrocarbons (e.g., gasoline) to prevent aspiration and severe esophageal injuries [24]. Induced emesis is reasonable within 1–2 h of toxicant ingestion; however, little evidence exists to support this narrow window of opportunity, and successful decontamination is described up to 4 h after toxicant ingestion if performed promptly [21, 24, 27, 42]. Gastric lavage could be considered if emesis induction has failed or is contraindicated, although the clinical benefit remains controversial [19, 21, 43, 44]. Additional IV fluid therapy should be considered in most cats with suspected poisoning and needs to be tailored to the individual toxicant and patient status [41]. It can support cardiovascular function and needs to be carefully titrated or even avoided with, for example, toxicants that can cause cerebral edema (e.g., bromethalin) or underlying heart disease. A toxicant-specific antidote was applied only for coumarin derivative poisonings (Vitamin K1). Elimination measures were performed almost twice as often as decontamination attempts, which was mainly IV lipid therapy (Lipofundin 20%, B. Braun, Melsungen, Germany) given to 60% of the cats, particularly to those with α-chloralose, permethrin, other biocides, NSAIDs, or THC poisoning.

Approximately 60% of cats were empirically treated with intravenous lipid emulsion (ILE), as no established protocols existed for managing feline poisoning cases involving hydrophobic agents and severe symptoms at that time. Along these lines, the attending clinician decided to treat, for example, α-chloralose poisoning cases with ILE (log P = 0.99). Particularly in light of possible treatment-associated complications (which also occurred), veterinarians should carefully consider routine ILE administration for hydrophobic toxicants for as long as sufficient evidence is lacking (e.g., log P < 1.0) [44, 45].

A survival rate of almost 90% and low mortality rates is consistent with other studies reporting an 86%, 83%, and 85% survival rate, respectively [3, 35, 36]. However, data for comparison are sparse in feline medicine. As most studies distinguish survival versus non-survival, this study differentiated natural death (2%) and euthanasia (10%) as lethal outcomes. Owners’ reasons for choosing euthanasia vary, and it is conceivable that some of these cats might have survived with additional therapy.

Toxicant-associated complications were more frequent (48%) than in a recent study with 39 cats (28%) [36]. Hypothermia was observed predominantly in rodenticides, hyperthermia due to local inflammation in the oral cavity, and pain triggered by didecyldimethylammonium chloride. Respiratory complications were most frequently noted, likely associated with cardiovascular depression and decreased oxygenation with hemorrhage or anemia. Gastrointestinal signs, including vomiting and superficial tongue lesions, can result from absorption and local effects of the toxicant, which may be prolonged. Nephrotoxicity was primarily associated with the ingestion of *Lilium* species, evident hemorrhage with coumarin derivatives, and a postictal comatose state with poisonings that did not result in hemorrhage. The hepatic metabolization of several toxicants can contribute to the development of liver failure.

We acknowledge the limitation of this study being retrospective in nature, carrying some risk of bias or lack of information. A standard assessment of the neurological status (e.g., Glasgow coma scale) was not utilized, and respiratory rates were mostly reported if abnormal. Laboratory trends were available from only a few patients. Retrospectively evaluating the likelihood of poisoning is challenging, and toxicological screening was rarely performed. Conclusions from suspected or “unknown” poisonings are generally limited and remain speculative for some clinical signs and treatment options.

## Conclusion

This study reviewed 166 feline cases of confirmed or suspected poisoning. Although the causative toxicant remained unidentified in most cases, rodenticides were the most common. Affected cats presented predominantly with neurological signs, reduced general condition, and/or hypothermia. A high survival rate was recorded; however, poisonings can present a life-threatening, serious situation requiring immediate and intensive veterinary care.

## Authors’ Contributions

CM: Conception and design of the study, analysis and interpretation of data, table preparation, and drafted the manuscript. RMH: Critical revision, interpretation of data, and drafted the manuscript. DK: Analysis and interpretation of data and acquisition of data. RD: Revision of the manuscript, table preparation, analysis and interpretation of data. All authors have read, reviewed, and approved the final manuscript.
